# Low dose radiation regulates BRAF-induced thyroid cellular dysfunction and transformation

**DOI:** 10.1186/s12964-019-0322-x

**Published:** 2019-02-13

**Authors:** Neha Kaushik, Min-Jung Kim, Nagendra Kumar Kaushik, Jae Kyung Myung, Mi-Young Choi, Jae-Hyeok Kang, Hyuk-Jin Cha, Cha-Soon Kim, Seon-Young Nam, Su-Jae Lee

**Affiliations:** 1 Department of Life Science, Research Institute for Natural Sciences, Hanyang University, Seoul, 04763 Republic of Korea; 2 Laboratory of Radiation Exposure and Therapeutics, National Radiation Emergency Medical Center, Korea Institute of Radiological and Medical Sciences, Seoul, South Korea; 3 Plasma Bioscience Research Center, Applied Plasma Medicine Center, Department of Electrical and Biological Physics, Kwangwoon University, Seoul, 01897 Republic of Korea; 4 Department of Radiation Pathology, Korea Cancer Center Hospital, Seoul, South Korea; 5 College of Pharmacy, Seoul National University, Seoul, South Korea; 6 Department of Preventive Medicine, College of Medicine, Dongguk University, Gyeongju, 38066 Korea; 7 Radiation Health Institute, Korea Hydro and Nuclear Power Co. Ltd, Seoul, South Korea; 8 Laboratory of Molecular Biochemistry, Department of Life Science, Hanyang University, 17 Haengdang-Dong, Seongdong-Ku, Seoul, 04763 South Korea

**Keywords:** Low dose radiation, LDR, Thyroid cancer, Paired-box domain 8, *PAX8*, miR-330-5p, Thyroglobulin, *TG*

## Abstract

**Background:**

The existence of differentiated thyroid cells is critical to respond radioactive iodide treatment strategy in thyroid cancer, and loss of the differentiated phenotype is a trademark of iodide-refractive thyroid disease. While high-dose therapy has been beneficial to several cancer patients, many studies have indicated this clinical benefit was limited to patients having *BRAF* mutation. *BRAF*-targeted paired box gene-8 (*PAX8*), a thyroid-specific transcription factor, generally dysregulated in *BRAF*-mutated thyroid cancer.

**Methods:**

In this study, thyroid iodine-metabolizing gene levels were detected in *BRAF*-transformed thyroid cells after low and high dose of ionizing radiation. Also, an mRNA-targeted approach was used to figure out the underlying mechanism of low (0.01Gyx10 or 0.1Gy) and high (2Gy) radiation function on thyroid cancer cells after *BRAF*^*V600E*^ mutation.

**Results:**

Low dose radiation (LDR)-induced *PAX8* upregulation restores not only *BRAF*-suppressive sodium/iodide symporter (NIS) expression, one of the major protein necessary for iodine uptake in healthy thyroid, on plasma membrane but also regulate other thyroid metabolizing genes levels. Importantly, LDR-induced *PAX8* results in decreased cellular transformation in *BRAF-*mutated thyroid cells.

**Conclusion:**

The present findings provide evidence that LDR-induced *PAX8* acts as an important regulator for suppression of thyroid carcinogenesis through novel *STAT3*/miR-330-5p pathway in thyroid cancers.

**Electronic supplementary material:**

The online version of this article (10.1186/s12964-019-0322-x) contains supplementary material, which is available to authorized users.

## Background

Recently activating somatic mutations in the *BRAF* proto-oncogene has been discovered in various malignancies, such as in melanoma (60–70% of cases) [1, 2], colon cancer (10%) [3, 4] including thyroid cancer (35–70%) [5, 6]. Thyroid tumors are the most frequent neoplasms of the endocrine system [7]. Well-differentiated thyroid carcinomas account for > 85% of all thyroid cancers including papillary and follicular carcinomas. In early 2003, BRAF mutations were reported in thyroid cancer with an occurrence ranging from 25 to 42% [8]. Papillary thyroid carcinoma (PTC) and anaplastic thyroid carcinoma (ATC) have only been reported with these mutations [8], however it has not been identified in follicular thyroid carcinoma (FTC), or benign thyroid adenomas [9]. Among all thyroid subtypes, PTC is the most prevalent and have an aggressive behavior [10], while undifferentiated anaplastic thyroid carcinoma accounts for 3 to 5% of all thyroid cancers [11].

BRAF is a serine/threonine kinases belongs to the RAF family. RAF proteins are part of the RAF-MEK-ERK pathway [mitogen activated protein (MAP)/extracellular signal-regulated kinase (ERK) kinase], a prominently conserved signaling component in eukaryotes. Once activated through binding to RAS in its GTP-bound state, RAF kinases phosphorylate MEK, thereby phosphorylates and activates ERK [12]. Activation of BRAF has originated as the most prevalent oncogenic mutation in thyroid carcinoma [5, 6, 13]. A trans-version from thymine to adenine (T1799A), leading to a Glu for Val substitution at residue 600 (V600E), accounts for > 92% of BRAF mutations in thyroid carcinomas [9]. Consistent with a pivotal role in thyroid cancer initiation, BRAF^V600E^ mutation has been identified in microcarcinomas [9], and it was revealed to induce transformed features in thyroid follicular cells in culture conditions [14]. A recent report suggests that ultraviolet radiation accelerates BRAF-driven melanogenesis by targeting TP53 [15]. Given that above well-characterized role of BRAF mutations prompted us to explore the functions of low dose of ionizing radiation (LDR) in BRAF-mediated cellular transformation in thyroid cancer cells. The advance of interventional radiology has attracted growing interest in the biological effect of LDR below 0.1Gy doses [16]. From our previous studies, it has become apparent that the LDR has the potential to block KRAS-driven cellular transformation [17] and metastatic cancer progression in breast cancer cells [18]. As, most of the thyroid patients can be cured with surgical treatment together with radioactive iodide, however BRAF-mutated thyroid cancer cells have lower expression of sodium/iodide symporter (*NIS*), thyroid transcription factors (*TTF-1 and TTF-2*), and thyroglobulin (*TG*) than those cells having wild-type (WT) *BRAF* [19], and are particularly refractory to radioiodine therapy [20]. The ability of differentiated thyroid cells to accumulate iodide is clinically highly relevant as it makes feasible for thyroid cancer patients to be treated with ablative doses of radioactive iodide after stimulation by thyroid-stimulating hormone [21]. Thus, the maintenance of the thyroid differentiated phenotype during tumor transformation has a critical impact in thyroid cancer patient’s survival [22]. PAX8, a member of the paired box (PAX) family of transcription factors are required for the maintenance of the thyroid differentiated phenotype [23]. Along with the other thyroid transcription factors TTF-2 and TTF-1, PAX8 is intricate in development of thyroid follicular cells as well as expression of thyroid-specific genes such as the *NIS*, and *TG* [24, 25]. These genes are essential for thyroid differentiation as they mediate the metabolism of iodide, leading to the synthesis of active thyroid hormone. One of the most important and well-established transcriptional targets of *PAX8* is *NIS*. This symporter is a central plasma membrane protein that mediates active iodide transport in the thyroid and other tissues [26]. In the healthy thyroid cells, NIS-mediated iodide uptake is the first step in thyroid hormone biosynthesis. Therefore, loss of the thyroid differentiated phenotype, particularly loss of *NIS* function, is one of the most important hallmarks of thyroid cancer progression.

The purpose of this study was to compare the iodine metabolizing genes expression profile induced by expression of BRAF^V600E^ in thyroid cells using LDR. Given that several miRNAs were associated with less differentiated tumors [27], here, we took a unified approach based on the existence of the miRNAs for LDR influence in thyroid carcinoma. Here, we investigated the restorability of thyroid specific genes expression by suppression of the miR-330-5p in PTC/ATC after LDR exposure. These findings could provide the positive role of LDR in thyroid cells expressing BRAF-mutant and to characterize those miRNAs involved in the alteration of genes essential for thyroid differentiation, namely *PAX8*.

## Materials and methods

### Chemicals and antibodies

Antibodies specific to pJAK1(Tyr1022/1023), JAK1, pJAK2(Tyr1007/1008), JAK2, STAT3 and BRAF were obtained from Santa Cruz Biotechnology, Inc. Antibodies to pSTAT3 (Tyr705) were purchased from Cell Signaling Technology. PAX8 antibody was purchased from Abcam (Seoul, Korea). NIS antibody was obtained from GeneTex, Inc., whereas 4,6-Diamidino-2-phenylindole (DAPI) and β-actin were obtained from Sigma, Korea. Anti-mouse or rabbit Alexa Fluor 488 and anti-rabbit or mouse Alexa Fluor 546 were purchased from Invitrogen. All controls, silencing RNA, micro RNAs mimics and inhibitor were bought from Genolution Pharmaceuticals, Inc., Korea. All primers including control and microRNA primers were designed and purchased from Macrogen, Korea. Hanks’ Balanced Salt solution (HBSS), HEPES, Ammonium cerium (IV) sulphate, Sodium arsenite (III) and sodium iodide were purchased from Sigma-Aldrich, Korea. The uptake buffer consisted of HBSS supplemented with HEPES (10 mM final conc.) was freshly prepared prior to assay.

### Cell culture and transfection

Human thyroid normal follicular N-thy ori-3-1, BHP 10–3, SNU-80, BCPAP, 850-5C and SNU-790 thyroid cancer cell lines were kindly gifted by Dr. Min-Jung Kim (KIRAMS, Korea). 850-5C cells were cultured in Dulbecco’s modified Eagle’s medium (DMEM) with high glucose (Hyclone, Korea) whereas other cell lines were cultured in Roswell park memorial institute medium (RPMI-1640). Normal thyroid cells were supplemented with 20 mM glutamine (Gibco, Korea). All cell cultures were supplemented with 10% fetal bovine serum (Hyclone) and maintained at 37 °C in a 5% CO_2_ atmosphere. Cells were passaged every two-three day and often treated with plasmocin™ (Invivogen) to prevent mycoplasma contamination. We used all cell lines about 25–30 passages to perform our experiments. Cell transfection was accomplished in 75–85% confluent cells using Lipofectamine 2000 Reagent (Invitrogen, USA) according to the manufacturer’s protocol. BRAF^V600E^ virus supernatant was made by Min-Jung Kim (KIRAMS, Korea) and treated directly to normal thyroid cells at a confluency of 75–80%.

### Irradiation

Thyroid cells were plated in 60-mm cell culture dishes and irradiated with a 137Cs laboratory γ-irradiator (LDI-KCCH 137, Seoul, Korea) using same procedure as described in our report previously [18].

### Western blot analysis

For Western blotting analysis, cells were harvested and lysed for protein extraction using cell lysis buffer [40 mM Tris-HCl (pH 8.0), 120 mM NaCl, 0.1% Nonidet-P40] supplemented with protease inhibitors. Proteins samples were separated by SDS-PAGE and blotted onto nitrocellulose membranes (Amersham, IL), blocked in 5% skim milk for 1 h (h) at room temperature and incubated with the primary antibodies overnight at 4 °C: anti-BRAF (1:1000), and anti-β-actin (1:1000). The blots were developed using horseradish peroxidase-conjugated secondary antibodies at room temperature for 2 h and visualized using enhanced chemiluminescence (ECL) procedures.

### Real time PCR analysis

Cell samples RNA was extracted using the Trizol reagent (Ambion). Quantification of RNA was done using the NanoDrop™ Spectrophotometer according to the manufacturer’s protocol. All qRT-PCR reactions were determined using the KAPA SYBR FAST qPCR kit from KAPA Biosystems (Wilmington, MA, USA). Amplification reactions were carried out in a Rotor Gene Q (Qiagen, Korea), and results were expressed as the fold change calculated by the ΔΔCt method relative to the control sample. β-actin was used for normalization as a control. For miRNA analysis, U6 small nuclear RNA was used as a control to determine relative miRNA expression.

### Tissue samples and immunohistochemistry

Paraffin blocks and slides from 10 cases of PTC and 4 cases of normal (between 1999 and 2013) were recovered from the archives of the Korea Cancer Center Hospital. Tissue sections were deparaffinized, rehydrated, exposed to antigen retrieval in antigen retrieval buffer, incubated with 3% hydrogen peroxide and non-specific binding was blocked using bovine serum albumin. These sections were incubated with mouse monoclonal anti-PAX8 antibody (1:400; Abcam) overnight at 4 °C, and then incubated with biotinylated secondary antibody bound to a streptavidin–horseradish peroxidase complex. The bound antibody was distinguished using 3,3-diaminobenzidine and the sections were counterstained with hematoxylin, dehydrated and mounted. All sections were recorded by individual pathologists.

### Immunofluorescence

For immunofluorescence, cells were fixed with 4% paraformaldehyde and permeabilized with 0.1% Triton X-100 in PBS. Afterwards, cells were incubated with the PAX8 (anti-mouse, 1:200), NIS (anti-rabbit, 1:200) primary antibodies in a solution of PBS with 1% bovine serum albumin and 0.1% Triton X-100 at 4 °C overnight. Next day, staining was visualized using anti-rabbit or anti-mouse Alexa Flour 488 and anti-rabbit or anti-mouse Alexa Flour 546 antibodies, and cells nuclei were counterstained using 4,6-diamidino-2-phenylindole (DAPI; Sigma). Stained cells were imaged with a confocal fluorescence microscope (Nikon).

### Flow cytometry

To examine the PAX8 positive cell population with respected experiments, the LDR-exposed cells were fixed with 2% paraformaldehyde and permeabilized with 90% methanol as suggested by manufacturer’s protocol. Then, these cells were labeled with an anti-PAX8 antibody. A respective control was also prepared and tested for each sample. The percentage of PAX8 positive cells were determined using PE (Alexa-flour^488^) and was analyzed using a BD FACSVerse cytometer and the FACS suite software.

### Thyroid stimulating hormone receptor (TSHR) ELISA assay

For TSHR determination, cell culture supernatant/blood serum was collected as desired time points in each experiment using colorimetric TSH receptor ELISA kit (LS biosciences, LS-F12873). All assay steps were performed according to kit manual instructions. This assay utilizes an antibody specific for Human TSH coated on a 96-well plate. The intensity of the color is measured at 450 nm using an ELISA plate reader.

### In vitro iodine uptake assay

Briefly, three days after cell seeding in 96-well cell culture plate, BCPAP cells formed approximately 75–80% confluency and assay procedure was adopted from previous report [28].

### Soft agar and sphere formation assays

For cellular transformation studies, we performed soft agar colony assays and sphere forming assays. To observe anchorage independent growth, a cell suspension (2 × 10^4^ cells) was suspended in 0.4% agar in growth medium and seeded in triplicate in 60-mm dishes pre-coated with 0.8% agar in growth medium and incubated at 37 °C with 5% CO_2_. After 16 days, colonies were photographed and counted in five randomly chosen fields and expressed as means of representative of two independent experiments. For tumor sphere assays, cells were plated as single-cell suspension in ultralow attachment 6-well plates and grown in serum free DMEM/F12 medium supplemented with 20 μl ml^− 1^ B27 (Invitrogen), 20 ng ml^− 1^ EGF and 20 ng ml^− 1^ bFGF. Fresh media (300 μl) was added every 3 days. At day 7, tumor spheres were counted and photographed. All sphere and colony formation assays were performed in triplicate.

### Animal experiments

To make an orthotopic model of thyroid carcinoma, NSG male mice (8–10 weeks old) Jackson Laboratories, Bar Harbor, ME, USA) were used according to previously established method [29]. Housing and all experimental animal procedures were approved by the Institutional Animal Care and Use Committee (IACUC) of the Center for Laboratory Animal Sciences, the Medical Research Coordinating Center, and the HYU Industry-University Cooperation Foundation. We used stable N-thy ori-3-1 thyroid cells (Mock and BRAF-untreated and LDR-treated) for animal experiment and 2.5 × 10^5^ cells/mouse (right side of thyroid gland) times the number mice injecting. Mice were sacrificed 25 days after cell injection and serum plasma were collected for TSHR Elisa assay. Injected thyroid glands were also excised for further experiments.

### Statistical analysis

All experimental results are represented as the mean ± standard deviation (S.D.) of at least three independent tests. The statistical analysis was performed using the parametric Student’s *t*-test to check the significance levels. Levels of significance are indicated as *p < 0.05; **p < 0.01; and ***p < 0.001.

## Results

### BRAF mutation induces hypothyroidism in thyroid cancer

**Fig. 1:**
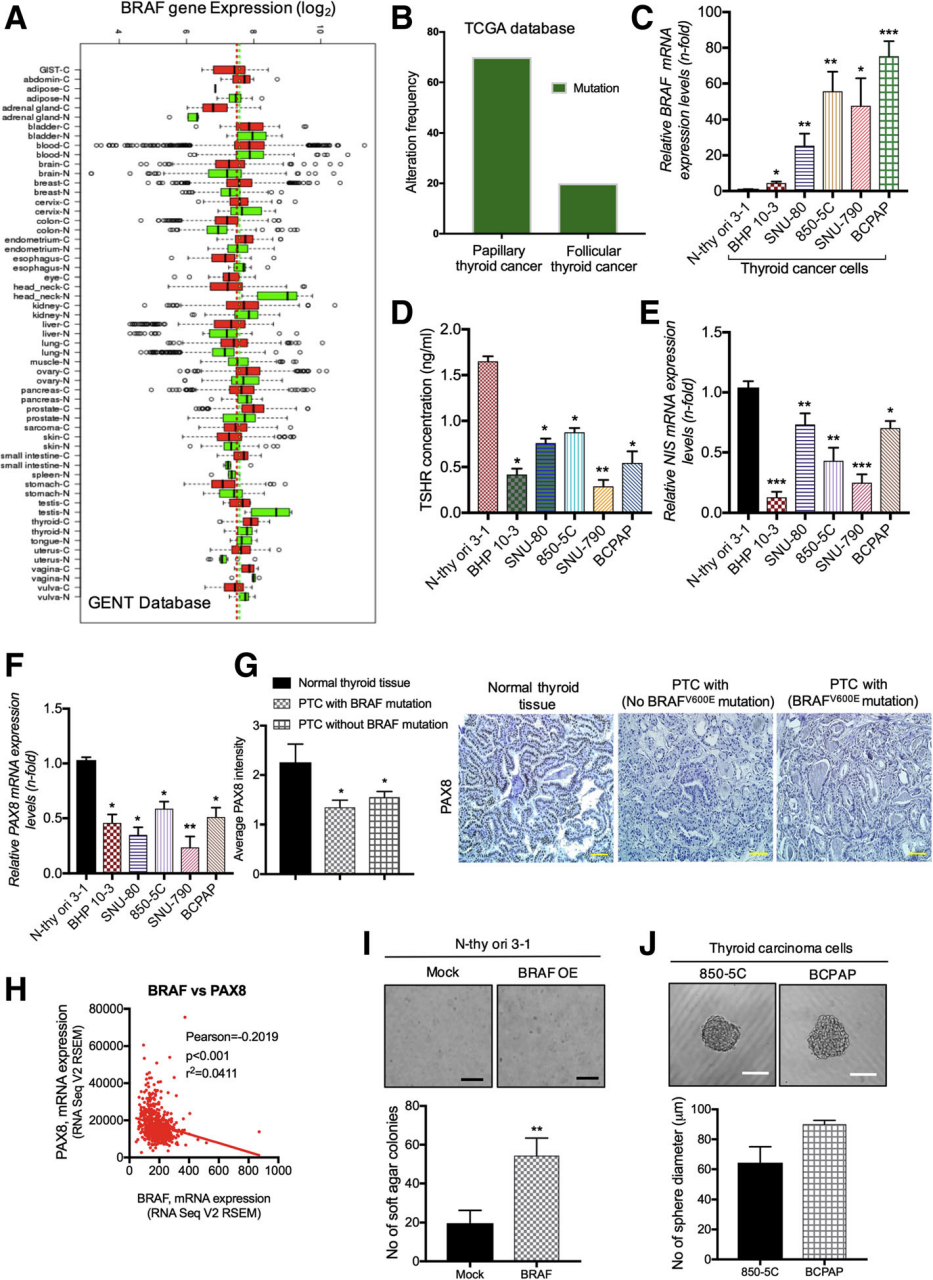
BRAF is highly expressed in thyroid cancer. **a** Analysis of BRAF expression in thyroid cancer among various cancer subtypes through publically available GENT database tool. **b** Evaluation of BRAF mutation frequency in papillary and follicular thyroid carcinoma using TCGA database. **c** Relative BRAF mRNA expression across normal follicular thyroid (N-thy ori-3-1), BRAF wild-type (BHP 10–3) and BRAF mutated-type (SNU-80, 850-5C, SNU-790, BCPAP) thyroid cancer cell lines. **d** ELISA assay for determination of TSHR levels in thyroid cells. **e**, **f** *NIS* and *PAX8* mRNA expression in several thyroid cell lines respectively. **g** Immunohistochemistry for *PAX8* expression in normal and PTC tumors as indicated in panels. **h** Co-relation studies of *BRA*F and *PAX8* mRNA expression using TCGA database. *PAX8* was negatively co-related with *BRAF* expression. **i**, **j** Soft agar colony and tumor sphere formation assay in BRAF^V600E^ mutated normal N-thy ori-3-1 transformed thyroid cells. Each experiment represents the mean of at least two independent set of experiments. *β-actin* was used as a control for normalization. Scale bar = 100 μm; *p < 0.05, **p < 0.001, and ***p < 0.0001

To determine the *BRAF* expression pattern in human thyroid tumors, we screened BRAF expression in normal and cancerous thyroid tissues using publically available GENT database [30]. Database profiling revealed that thyroid tumors demonstrated increased *BRAF* expression compared to normal thyroid tumors (Fig. 1a). Moreover, the cancer genome atlas (TCGA) analysis [31] shows high BRAF mutation rate in PTC compared to FTC tumors (Fig. 1b). Since BRAF^V600E^ accounts for > 85% of BRAF mutations in thyroid carcinomas as described previously [9], we evaluated the *BRAF*^*V600E*^ expression in human thyroid cancer cells. As shown in Fig. 1c, *BRAF*^*V600*E^ mRNA levels were significantly higher in ATC (SNU-80, 850-5C) and PTC (SNU-790, BCPAP) as compared to wild type (BHP 10–3) and normal thyroid follicular epithelial cells (N-thy ori-3-1). It has been shown earlier that lower levels of serum TSH owing to severe hypothyroidism in Braf^V600E^-induced thyroid cell models [32, 33]. Accordingly, when we analyzed TSHR levels in PTC and ATC cells, we observed that TSHR levels were lower in these cells as compared to normal thyroid follicular N-thy ori-3-1 cells (Fig. 1d). High levels TSHR signaling requires to upregulate *NIS* expression and iodide uptake in differentiated thyroid cancer cells [34]. We sought to investigate the *NIS* expression in BRAF mutated thyroid cells. Likewise, BRAF^V600E^ mutated as well as wild type thyroid cancer cells demonstrated relatively low expression of *NIS* as well as *PAX8* (direct regulator of NIS) compared to normal cells (Figs. 1e & f). In addition, PTC tumors shows lower *PAX8* expression which have BRAF^V600E^ mutation (Fig. 1g). TCGA database further revealed that *PAX8* is negatively co-related in BRAF mutated thyroid tumors (Fig. 1h). To detect BRAF mutation consequences on thyroid cells, we transformed normal N-thy ori-3-1 cells with BRAF^V600E^ mutation expression vector. A significant induction in thyroid cellular transformation was observed in N-thy ori-3-1 transformed cells as visualized by soft agar colony formation and sphere forming assays (Figs. 1i-j). These studies confirmed that BRAF^V600E^ mutation suppresses the iodine metabolizing gene levels and induce cellular transformation in thyroid carcinoma cells, prompting us to explore the role of LDR in the BRAF-induced cellular transformation at initiation levels.

### LDR inhibits BRAF-driven cellular transformation and rescues repression of iodine-metabolizing gene expression responsible for thyroid cancer initiation

**Fig. 2:**
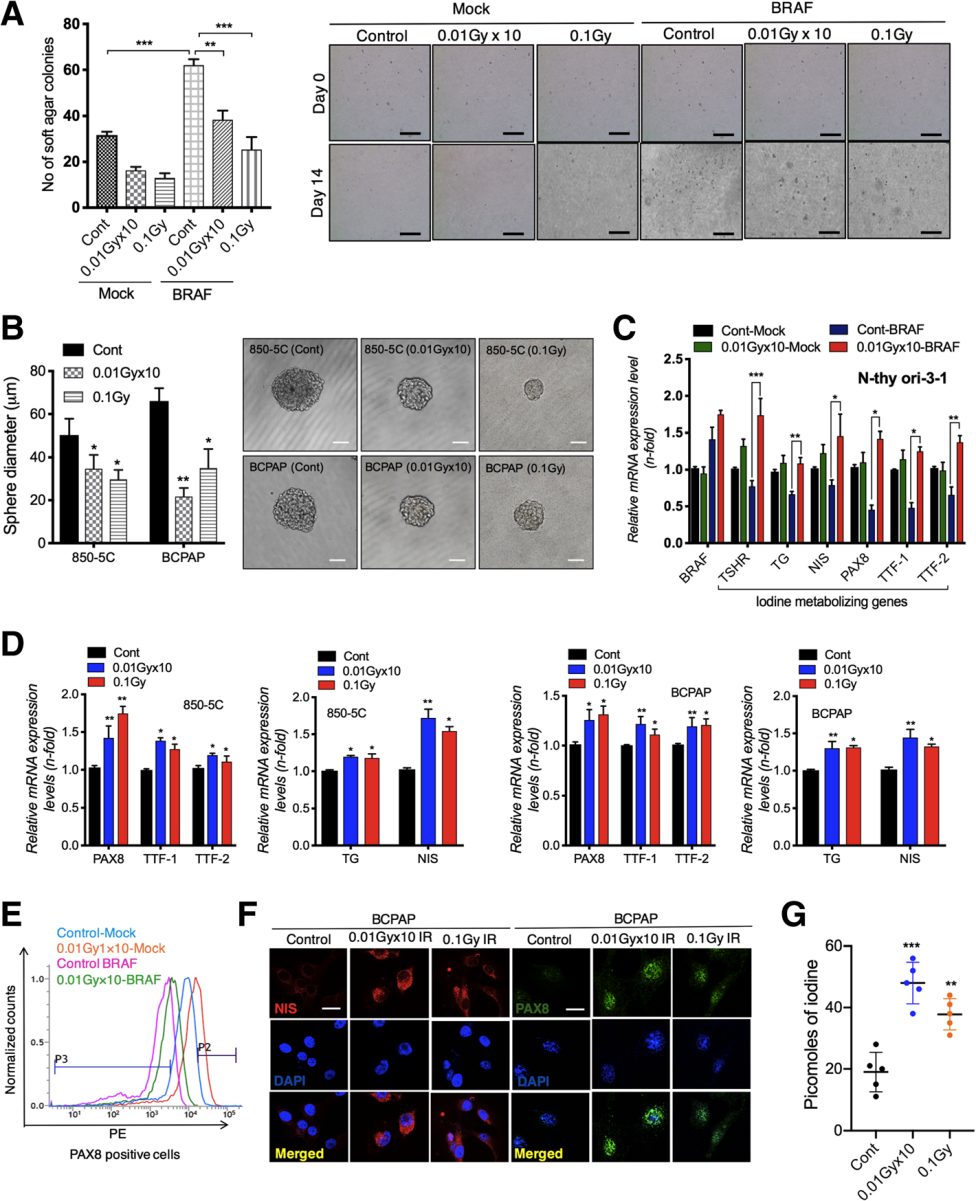
LDR blocks cellular transformation through upregulating iodine-metabolizing gene expression in BRAF-transformed thyroid cells. **a** Soft agar colony formation in BRAF^V600E^ transformed N-thy ori-3-1 cells after fractionated (0.01Gy× 10) and single dose (0.1Gy) of LDR. **b** Quantification of tumor sphere formation in 850-5C and BCPAP thyroid cancer cells after fractionated (0.01Gy × 10) and single dose (0.1Gy) of LDR. **c** Relative mRNA expression of thyroid iodine metabolizing genes in BRAF^V600E^ transformed N-thy ori-3-1 cells after fractionated (0.01Gy × 10) dose of radiation. **d** Relative mRNA expression of thyroid iodine metabolizing genes in 850-5C and BCPAP cells after fractionated (0.01Gyx10) and single dose (0.1Gy) of LDR. **e** Flow cytometry analysis of PAX8 positive cells in BRAF^V600E^ transformed N-thy ori-3-1 cells after fractionated (0.01Gyx10) dose of radiation. **f** Immunofluorescence for *NIS* and *PAX8* expression levels in 850-5C and BCPAP thyroid cancer cells after fractionated (0.01Gyx10) and single dose (0.1Gy) of LDR. **g** A non-radioactive iodine assay was performed in BCPAP thyroid cancer cells after fractionated (0.01Gyx10) and single dose (0.1Gy) of LDR. *β-actin* was used as a control for normalization. Scale bar = 50 μm; *p < 0.05, **p < 0.001, and ***p < 0.0001

We have previously shown that LDR have potential to reduce cancer progression at fractionated (1cGyx10) or single doses (0.1Gy or 10cGy) [17, 18]. In accordance, we confirmed that LDR diminishes the growth of 850-5C, BCPAP as well as N-thy ori-3-1 BRAF^V600E^ transformed thyroid cancer cells at both doses (Additional file 1: Figure S1A). Therefore, we decided to use these doses to interrogate whether LDR is capable to reduce cellular transformation in BRAF transformed thyroid cells. Interestingly, LDR significantly suppresses colony formation in N-thy ori-3-1 cells at both doses fractionated as well as single doses (Fig. 2a). A comparable effect was also seen in 850-5C and BCPAP thyroid cancer cells after LDR exposure as confirmed by sphere forming assays (Fig. 2b). These findings lead us to investigate whether LDR can increase expression of iodine-metabolizing genes in normal N-thy ori-3-1 cells after BRAF^V600E^ mutation responsible for thyroid carcinogenesis. Interestingly, LDR restores the *TSHR*, *TG*, *NIS*, *PAX8*, *TTF-1* and *TTF-2* mRNA levels in normal thyroid BRAF-transformed cells (Fig. 2c), whereas HDR was fail to increase expression of these genes N-thy ori-3-1 BRAF transformed cells at a dose of 2Gy HDR (Additional file 1: Figure S2A). Similar results were obtained in in BCPAP thyroid cancer cells at a dose of 2Gy HDR (Additional file 1: Figure S2B). Comparable observations were also seen on thyroid iodine-metabolizing genes in case of 850-5C (ATC) and BCPAP (PTC) cell lines after LDR exposure (Fig. 2d). Since PAX8 is critical for NIS targeting, responsible for iodine uptake, we performed FACS analysis to check PAX8 positive population. A significant increase in PAX8 positive cells were detected in LDR exposed BRAF-transformed cells when compared to BRAF transformed cells without LDR treatment (Fig. 2e). Consistent with these findings, we observed that increased *PAX8* (nuclear expression) improves *NIS* (membrane localization) expression in LDR-exposed BCPAP cells (Fig. 2f). Interestingly, iodine uptake assay showed that LDR can restore iodine uptake in BCPAP cancer cells as seen by Fig. 2g. Taken together, these data support that LDR actively restores BRAF-dependent repression of thyroid iodine metabolizing genes in thyroid cells at the initiation phase of cancer progression.

### PAX8 upregulation is critical in decreased cellular carcinogenesis

**Fig. 3:**
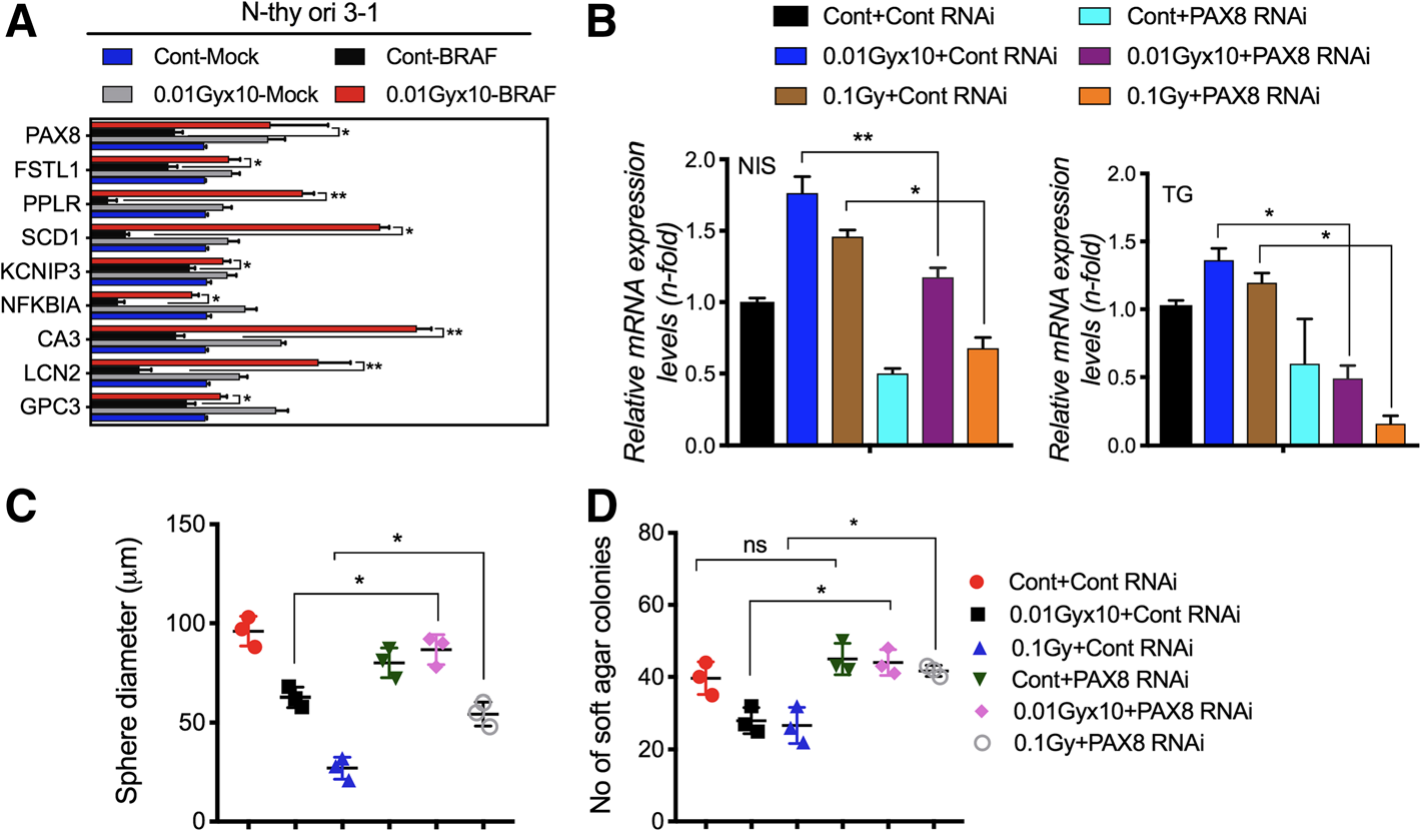
PAX8 suppressed thyroid carcinogenesis after LDR exposure. **a** Analysis of PAX8 target signature genes in BRAF^V600E^ transformed N-thy ori-3-1 cells after fractionated (0.01Gyx10) dose of radiation. **b** qPCR analysis of *NIS* and *TG* expression in LDR-treated 850-5C thyroid cancer cells after PAX8 silencing. **c**, **d** Sphere and colony formation was determined in 850-5C cancer cells in same conditions as shown in B. *β-actin* was used as a control for normalization. *p < 0.05, **p < 0.001, and ***p < 0.0001

As mentioned earlier, PAX8 is critical for the cellular differentiation, being a direct mediator of the thyroid-specific expression of the genes expressed in the thyroid cell type [23]. We questioned whether LDR can also target PAX8 signature genes upregulation as described previously [35]. Real time PCR analysis showed that LDR treatment potently upregulated the *PAX8* target signature genes in BRAF^V600E^ transformed N-thy ori-3-1 cells (Fig. 3a). Since, we observed above that LDR increases *PAX8* expression effectively, we wanted to further examine whether PAX8 upregulation is critical in maintenance of thyroid functional state. To this end, we checked *NIS* and *TG* expression after PAX8 knock-down in LDR-exposed cells, which are chief determinants of thyroid iodine metabolizing. Data showed that PAX8 silencing abolished the effect of LDR on *NIS* and *TG* upregulation in 850-5C cancer cells (Fig. 3b). Likewise, PAX8 knockdown also harbored LDR-mediated suppression of BRAF-induced cellular transformation in 850-5C cancer cells as observed by sphere forming and soft agar colony forming assays (Figs. 3c & d). These observations clearly suggest that LDR could effectively inhibit the cellular transformation of BRAF-mutated cells potentially through the induction of *PAX8* and its target genes.

### Loss of miR-330-5p leads to PAX8 upregulation by LDR and improve NIS/TG expression

**Fig. 4:**
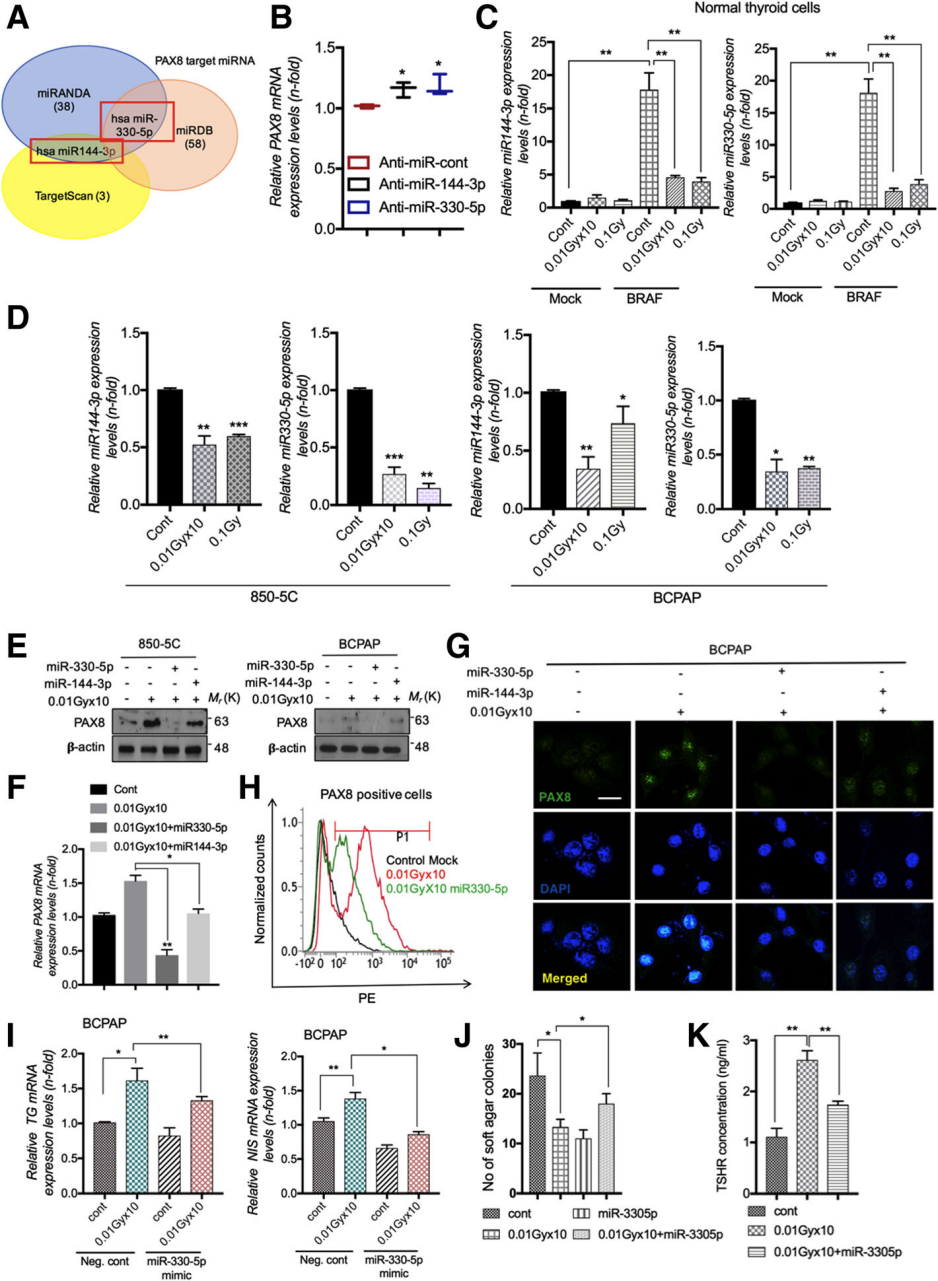
miR-330-5p targets PAX8 and is downregulated by LDR. PAX8 suppressed thyroid carcinogenesis after LDR exposure. **a** Venn diagram showing the PAX8 targeting miRNAs predicted by miRNA prediction tools as mentioned in panels. **b** Determination of *PAX8* mRNA after anti-miR-144-3p and anti-miR-330-5p treatment in 850-5C thyroid cancer cells. **c**, **d** qPCR analysis of miR-144-3p and miR-330-5p levels in BRAF^V600E^ transformed N-thy ori-3-1, BRAF-mutated 850-5C and BCPAP thyroid cancer cells after fractionated (0.01Gyx10) and single dose (0.1Gy) of LDR respectively. **e**, **f** miR-144-3p and miR-330-5p mimics were treated in LDR-exposed 850-5C and BCPAP cells and PAX8 levels were detected by western blot and qPCR analysis respectively. **g** Immunofluorescence of PAX8 levels in LDR-treated BCPAP thyroid cancer cells after miR-144-3p and miR-330-5p mimics transfection. **h** FACS analysis for PAX8 expression in LDR-treated BCPAP cells after miR-330-5p transfection. **i**, **j** *TG*, *NIS* mRNA levels and soft agar colony formation was analyzed in LDR-treated BCPAP thyroid cancer cells after miR-330-5p mimetics transfection. **k** TSHR levels were detected by ELISA assay in cell supernatants of LDR-treated BCPAP thyroid cancer cells in similar conditions as used in H. *β-actin* was used as a control for normalization. *p < 0.05, **p < 0.001, and ***p < 0.0001

To carry out an in-depth analysis of the LDR-mediated PAX8 induction, we screened putative PAX8 targeting microRNAs (miRNAs) using online available prediction tools TargetScan [36] and miRDB [37] and miRANDA [38]. These different tools have been developed for prediction of miRNA-mRNA interactions. Most well-characterized function of miRNAs to downregulate gene expression by binding to complementary sites within transcript sequences and suppress their translation and eventually stimulate their degradation [39]. To determine whether LDR may drive the PAX8 restoration in thyroid carcinoma cells through the inhibition of miRNAs, we assessed PAX8 targeting miRNAs as above mentioned techniques. We found that miR-144-3p and miR-330-5p were most prominent candidate for PAX8 targeting (Fig. 4a). To confirm miRNAs identified by prediction tools, we checked further PAX8 levels in 850-5C thyroid carcinoma cells after miR-144-3p and miR-330-5p inhibitor treatment. PAX8 levels were significantly higher in 850-5C after these miRNAs silencing (Fig. 4b & Additional file 1: Figure S3A), suggesting the involvement of these miRNAs in LDR-induced PAX8 expression. In BRAF^V600E^ transformed N-thy ori-3-1 thyroid cells, the expression of miR-144-3p and miR-330-5p were greatly reduced as compared to non-irradiated one (Fig. 4c). Furthermore, the expression of these miRNAs was also markedly decreased in the 850-5C and BCPAP cancer cells after LDR treatment (Fig. 4d), whereas HDR stimulated marked induction in miR-144-3p and miR-330-5p expression in both cell types (Additional file 1: Figure S3B). Exogenous treatment with miR-330-5p mimetics abolished the effect of LDR on PAX8 induction in 850-5C and BCPAP cells more prominently, however this effect was negligible in case of miR-144-3p in both cells (Figs. 4e, f & Additional file 1: Figure S3C). As expected, immunofluorescence staining showed the similar effects on BCPAP cells after miR-330-5p and miR-144-3p overexpression in response to radiation (Fig. 4g). In addition, FACS analysis confirmed that LDR-mediated PAX8 induction was interrupted by miR-330-5p mimics treatment in BCPAP cancer cells (Fig. 4h). Of note, ELISA assay shows recovered TSHR levels in normal N-thy ori-3-1 transformed cells after LDR (Additional file 1: Figure S1B). Therefore, consistent with these studies, miR-330-5p overexpression abolished the effect on *TG*, *NIS*, colony formation and TSHR levels in BCPAP cells in response of LDR (Figs. 4i-k). Hence, we hypothesized that LDR-mediated induction of iodine-metabolizing genes could be explained by the inhibition of miRNA pathway in thyroid cancer cells.

### STAT3 acts as an upstream regulator for miR-330-5p essential for thyroid malfunction decreased by LDR

**Fig. 5:**
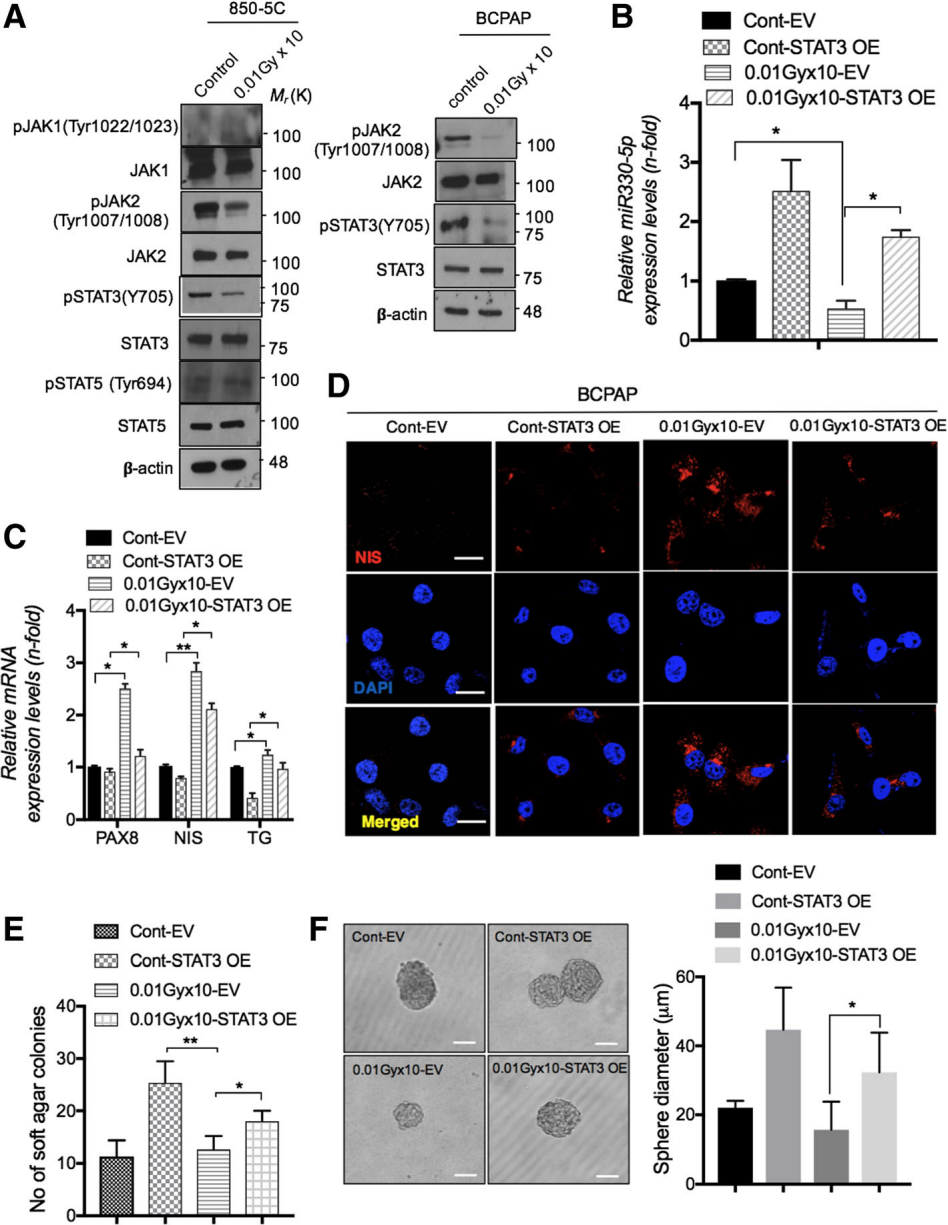
LDR reduces activation of JAK2/STAT3 and downregulates miR-330-5p levels to reduce cellular transformation. **a** Phosphorylation status of JAK1, JAK2, STAT3, STAT5 was observed by western blot analysis in 850-5C and BCPAP thyroid cancer cells. **b**, **c** qPCR analysis for miR-330-5p and thyroid specific gene (*PAX8*, *NIS* and *TG*) levels in STAT3 overexpressing BCPAP thyroid cancer cells after LDR treatment. **d** Immunocytochemistry for NIS expression in STAT3 overexpressing BCPAP cells after LDR treatment. **e**, **f** Soft agar colonies and sphere formation was determined in BCPAP cells in same conditions as shown in D. Scale bar = 100 and 50μm. *β-actin* was used as a control for normalization. *p < 0.05, **p < 0.001, and ***p < 0.0001

Since, recently Lesinski suggested that targeting STAT3 could overcome BRAF-resistance tumors [40], to identify signaling mechanism underlying LDR deregulated miRNAs, we first checked phosphorylation status of JAK/STAT pathway. We found that JAK2 and STAT3 were reduced after LDR treatment in both 850-5C and BCPAP cancer cells (Fig. 5a). Next, to confirm the role of STAT3 in LDR-decreased miRNAs expression, we overexpressed BCPAP cells with *STAT3* expression vector. Noticeably, forced expression of *STAT3* blocked the LDR-effect on miR-330-5p downregulation in BCPAP thyroid cells (Fig. 5b). Subsequently, *PAX8*, *NIS* and TG were also reduced after *STAT3* overexpression in BCPAP cells (Figs. 5c & d). Finally, we confirmed these findings on cellular transformation studies in thyroid cells. We found that exogenous expression of *STAT3* abrogated the effect of LDR on thyroid carcinogenesis as shown by soft agar colony formation and sphere formation assays (Figs. 5e & f) in BCPAP cells. We finally wanted to investigate how LDR can suppress STAT3 activation in thyroid cancer cells. While the suppressor of cytokine signaling (SOCS) proteins are known as negative regulators of JAK/STAT signaling pathways [41], we screening SOCS family proteins in LDR-exposed BCPAP thyroid carcinoma cells. Among all tested SOCS members, *SOCS3* and *SOCS4* were notably increased in response to radiation (Additional file 1: Figure S3D). Taken together, these data strongly support a mechanism by which LDR actively represses STAT3-dependent miR-330-5p in BRAF-mutated thyroid cells, thereby upregulating PAX8 and related target genes.

### Expression of thyroid metabolizing genes are dramatically restored in BRAF-mutant mice model

**Fig. 6:**
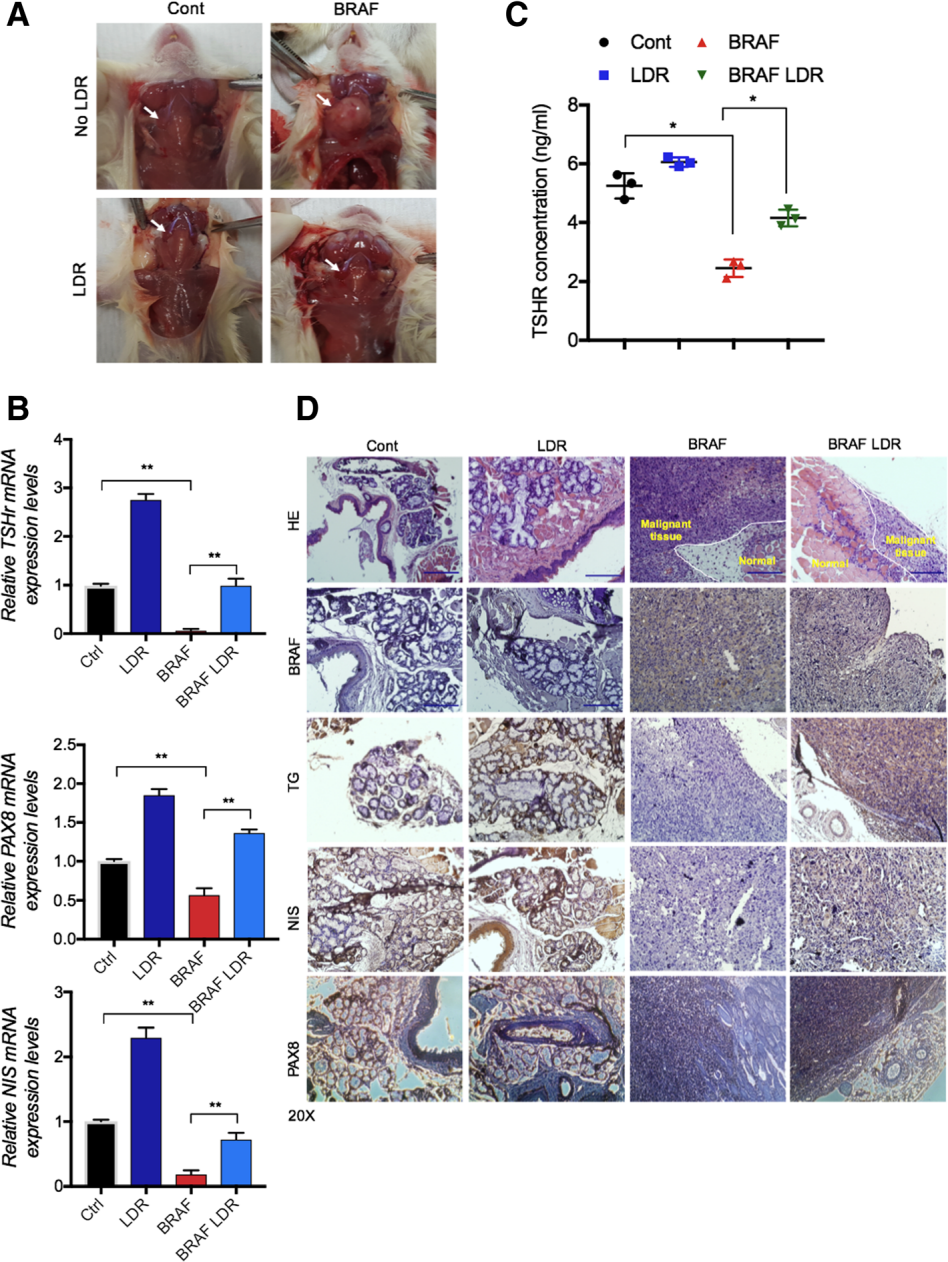
LDR reduces thyroid carcinogenesis in Orthotopic mice xenografts. **a** Pictorial presentation of thyroid orthotopic mice models (Untreated and LDR-treated). **b** qRT-PCR analysis of TSHR, NIS and PAX8 mRNA expression in LDR-treated and untreated thyroid gland tissues. **c** TSHR levels were detected by ELISA assay in blood serum of control and LDR-treated BCPAP thyroid cancer cells. **d** Hematoxylin Eosin (HE)/IHC images of thyroid gland sections for NIS, TG, BRAF and PAX8 expression

As in vitro experiments revealed that LDR can improve thyroid metabolism regulating genes in thyroid cancer cells, and these cells also reduce transformation process. We then considered whether exposure of LDR might suppress carcinogenesis process in thyroid xenograft mice models. To this end, we made an orthotopic thyroid mice model and injected BRAF^V600E^ thyroid cells in thyroid gland of mice. Upon sacrifice, we detected that thyroid gland size is much smaller in LDR exposed BRAF-mutated group compared to only BRAF injected group (Fig. 6a). Remarkably, levels of TSHR, PAX8 and NIS also increased in LDR-exposed BRAF mice group (Figs. 6b-d). These data further establish LDR acts as a suppressor for thyroid carcinogenesis in mice and, thereby indicate a promising candidate in radiation biology.

## Discussion

Generally, the diagnosis for PTCs is suitable unless the tumor presents a well or poorly differentiated phenotype. It is believed that dedifferentiation is correlated with molecular alterations of proteins that permit the thyrocytes to concentrate the iodine, which render the tumor refractive to radioiodine treatment [22]. Aberrant activation of BRAF^V600E^ mutation is accompanying with the loss of radioiodine uptake and therapy failure in PTC. Reliably, BRAF^V600E^ mutation is highly predominant (80–90%) in recurrent radioiodine-refractory PTC/ATC patients [42, 43], as comparison with the lower occurrence of BRAF^V600E^ mutation (40%) in primary PTC [9]. Several studies have demonstrated an association of BRAF^V600E^ mutation with reduced expression of thyroid iodide-metabolizing genes namely *NIS*, *TSHR*, *TG* in thyroid cancer and induces hypothyroidism [19, 44]. Moreover, Liu et al. showed that BRAF^V600E^ is directly involved in impairment of the expression of almost these genes using a MEK inhibitor [33]. Consistent with this studies, we also identified that *PAX8* has lower expression in PTC and ATC patients having BRAF^V600E^ mutation (Fig. 1g).

Molecular events leading to the loss of thyroid differentiation in thyroid cancer draws much attention, especially loss of *NIS* expression. NIS is required for the active transport of iodide into the thyroid cells, and for the diagnosis and therapeutic management of thyroid cancer patients treated with radioiodine. We determine for the first time that LDR critically enhanced *PAX8* expression levels in PTC and ATC expressing BRAF^V600E^ mutant. PAX8 is a transcription factors that controls the transcription of *NIS*, is essential for the thyroid gland development and for maintaining the differentiated state in the normal thyroid gland [45]. We also show that PAX8 modulated *NIS* expression in thyroid cancer after LDR exposure in thyroid cells. PAX8 overexpression facilitate *NIS* expression on plasma membrane and improve its localization (data not shown). To figure out how the LDR-mediated induction of *PAX8* involved in thyroid-specific activation of the *NIS* and *TG* gene, we took advantage of the miRNA-based approach. Interestingly, the results obtained with the prediction online tools indicate that the miR-144-3p and miR-330-5p containing the already known PAX8 targeting sites could be involved in LDR function. In real time PCR assays, LDR treatment downregulated both miRNAs expression very efficiently, whereas the HDR responded poorly to these miRNAs, showing an activation. On the contrary, miR-330-5p mimetics interfered with the effect on LDR on *NIS*, *TG* and colony formation in PTC (Figs. 4i-j). As miRNAs expression decreases in response to LDR rather than HDR, we identified upstream molecule which can be affected through LDR. The results emphasize that JAK2/STAT3 activation was harbored after LDR in PTC/ATC (Fig. 5a). The STAT3 overexpression state contradicted LDR function in maintenance of PAX8, *NIS*, *TG* gene expression that may contribute to cellular transformation (Figs. 5c & f). Despite the sensitivity of LDR to BRAF-induced cellular transformation in thyroid cells, their BRAF expression levels were not downregulated by LDR from those detected in BRAF^V600E^ cells. It could be assumed that some other pathway is intricate in STAT3 activation rather than BRAF phosphorylation. To resolve this question, we measured the SOCS family proteins, whose expression negatively regulate JAK/STAT signaling after LDR treatment. In doing so, we found that *SOCS3* and *SOCS4* were majorly induced in LDR-treated PTC cells (Additional file 1: Figure S3D). In addition, Kleiman et al., suggested that the marked reduction of TSHR co-operates with oncogenic BRAF induced dysregulation of thyroid development [32] and TSHR signaling is required for expression of a subset of thyroid-specific genes during development [46]. Our data indicated LDR effectively recovered TSHR levels in thyroid carcinoma cells (Additional file 1: Figure S1B), perhaps decreasing STAT3-miRNA axis route accounting for the high undifferentiated phenotype.

## Conclusion

**Fig. 7:**
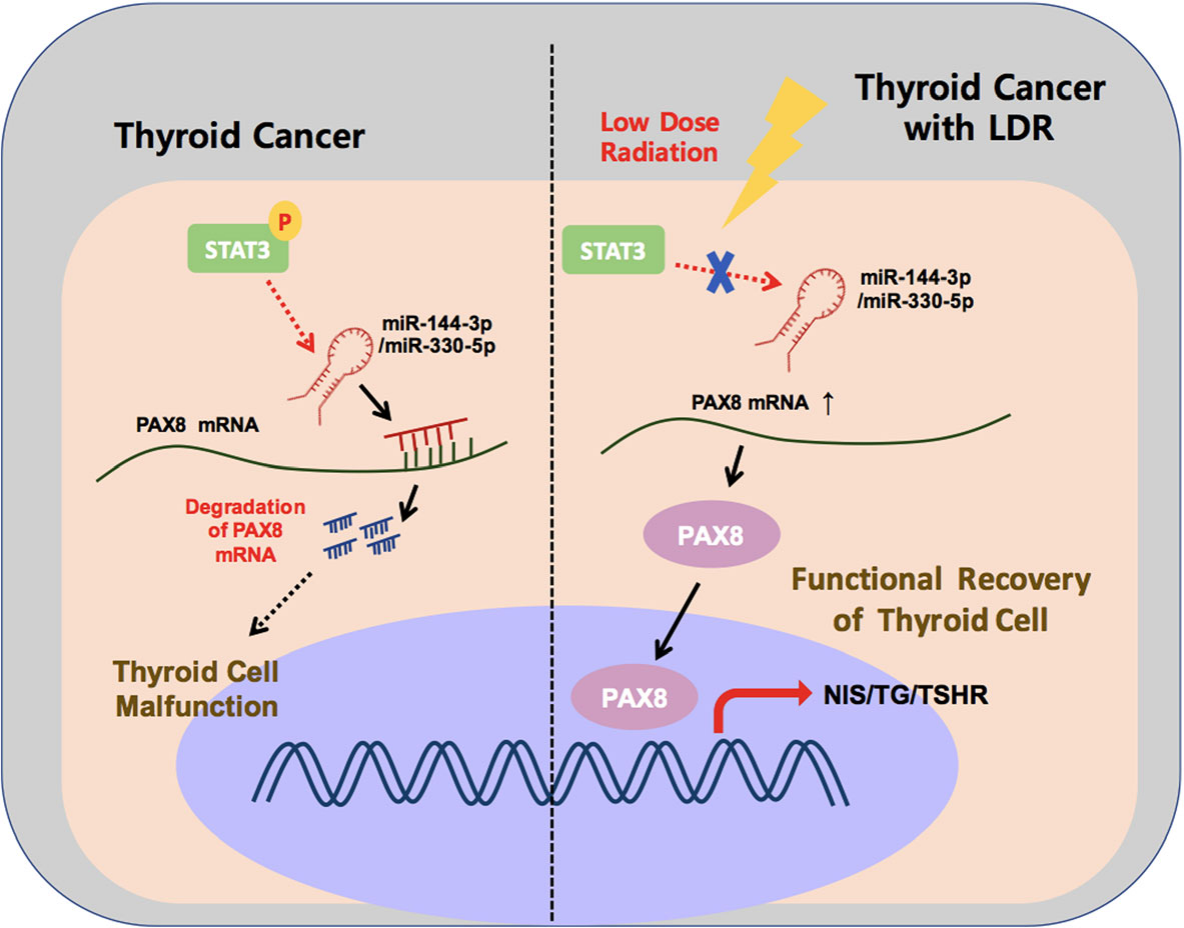
LDR suppresses the thyroid cancer carcinogenesis through STAT3-miR-330-5p axis. During thyroid cell malfunction, miR-144-3p/miR-330-5p degrades the PAX8 mRNA levels, thereby blocks its activity to NIS, TG and TSHR promoters. However, LDR treatment decreases the PAX8 targeting miRNAs expression and eventually recovers the PAX8 activity on its target gene promotes to improve thyroid cell function in cancer cells

In this study, we identified a signaling regulatory network in LDR-mediated PTC carcinogenesis suppression that is responsible in BRAF^V600E^-mutated thyroid cancer phenotypic transformation. Our studies characterized LDR can act as a repressor of BRAF induced STAT3-mediated miR-330-5p axis that eventually upregulates thyroid metabolizing genes expression through PAX8 induction, unveiling a regulatory circuit that defines the undifferentiated phenotype in PTC (Fig. 7). These studies provide LDR could be beneficial in alteration in these molecules that governs BRAF^V600E^-dependent cellular transformation in thyroid carcinoma cells and constitute potential new therapeutic targets for thyroid patients.

## Additional file

Additional file 1: **Figures S1-S3**. (DOCX 612 kb)

## Abbreviations

BRAF V600E: Amino acid substitution at position 600 in BRAF from a valine (V) to a glutamic acid (E)
BRAF: Serine/threonine-protein kinase B-Raf
ELISA: Enzyme-linked immunosorbent assay
FACS: Fluorescence-activated cell sorting
JAK2: Janus kinase 2
MEK-ERK: Mitogen-activated protein kinase kinase-Extracellular regulated kinases
SOCS: Suppressor of cytokine signaling proteins
STAT3: Signal transducer and activator of transcription 3

## References

[1] Brose MS, Volpe P, Feldman M, Kumar M, Rishi I, Gerrero R, Einhorn E, Herlyn M, Minna J, Nicholson A (2002). BRAF and RAS mutations in human lung cancer and melanoma. Cancer Res.

[2] Davies H, Bignell GR, Cox C, Stephens P, Edkins S, Clegg S, Teague J, Woffendin H, Garnett MJ, Bottomley W (2002). Mutations of the BRAF gene in human cancer. Nature.

[3] Rajagopalan H, Bardelli A, Lengauer C, Kinzler KW, Vogelstein B, Velculescu VE (2002). Tumorigenesis: RAF/RAS oncogenes and mismatch-repair status. Nature.

[4] Yuen ST, Davies H, Chan TL, Ho JW, Bignell GR, Cox C, Stephens P, Edkins S, Tsui WW, Chan AS (2002). Similarity of the phenotypic patterns associated with BRAF and KRAS mutations in colorectal neoplasia. Cancer Res.

[5] Kimura ET, Nikiforova MN, Zhu Z, Knauf JA, Nikiforov YE, Fagin JA (2003). High prevalence of BRAF mutations in thyroid cancer: genetic evidence for constitutive activation of the RET/PTC-RAS-BRAF signaling pathway in papillary thyroid carcinoma. Cancer Res.

[6] Cohen Y, Xing M, Mambo E, Guo Z, Wu G, Trink B, Beller U, Westra WH, Ladenson PW, Sidransky D (2003). BRAF mutation in papillary thyroid carcinoma. J Natl Cancer Inst.

[7] Segura S, Ramos-Rivera G, Suhrland M (2018). Educational case: endocrine neoplasm: medullary thyroid carcinoma. Acad Pathol.

[8] Hundahl SA, Fleming ID, Fremgen AM, Menck HR (1998). A National Cancer Data Base report on 53,856 cases of thyroid carcinoma treated in the U.S., 1985-1995 [see commetns]. Cancer.

[9] Xing M (2005). BRAF mutation in thyroid cancer. Endocr Relat Cancer.

[10] Sherman SI (2003). Thyroid carcinoma. Lancet.

[11] Ragazzi M, Ciarrocchi A, Sancisi V, Gandolfi G, Bisagni A, Piana S. Update on anaplastic thyroid carcinoma: morphological, molecular, and genetic features of the Most aggressive thyroid Cancer. Int J Endocrinol. 2014. 10.1155/2014/790834 PMC4158294 25214840

[12] Tuveson DA, Weber BL, Herlyn M (2003). BRAF as a potential therapeutic target in melanoma and other malignancies. Cancer Cell.

[13] Soares P, Trovisco V, Rocha AS, Lima J, Castro P, Preto A, Maximo V, Botelho T, Seruca R, Sobrinho-Simoes M (2003). BRAF mutations and RET/PTC rearrangements are alternative events in the etiopathogenesis of PTC. Oncogene.

[14] Mitsutake N, Knauf JA, Mitsutake S, Mesa C, Zhang L, Fagin JA (2005). Conditional BRAFV600E expression induces DNA synthesis, apoptosis, dedifferentiation, and chromosomal instability in thyroid PCCL3 cells. Cancer Res.

[15] Viros A, Sanchez-Laorden B, Pedersen M, Furney SJ, Rae J, Hogan K, Ejiama S, Girotti MR, Cook M, Dhomen N, Marais R (2014). Ultraviolet radiation accelerates BRAF-driven melanomagenesis by targeting TP53. Nature.

[16] Mobbs SF, Muirhead CR, Harrison JD (2011). Risks from ionising radiation: an HPA viewpoint paper for Safegrounds. J Radiol Prot.

[17] Kim RK, Kim MJ, Seong KM, Kaushik N, Suh Y, Yoo KC, Cui YH, Jin YW, Nam SY, Lee SJ (2015). Beneficial effects of low dose radiation in response to the oncogenic KRAS induced cellular transformation. Sci Rep.

[18] Kaushik N, Kim MJ, Kim RK, Kumar Kaushik N, Seong KM, Nam SY, Lee SJ (2017). Low-dose radiation decreases tumor progression via the inhibition of the JAK1/STAT3 signaling axis in breast cancer cell lines. Sci Rep.

[19] Durante C, Puxeddu E, Ferretti E, Morisi R, Moretti S, Bruno R, Barbi F, Avenia N, Scipioni A, Verrienti A (2007). BRAF mutations in papillary thyroid carcinomas inhibit genes involved in iodine metabolism. J Clin Endocrinol Metab.

[20] Xing M (2007). BRAF mutation in papillary thyroid cancer: pathogenic role, molecular bases, and clinical implications. Endocr Rev.

[21] American Thyroid Association Guidelines Taskforce on Thyroid N, Differentiated thyroid C, Cooper DS, Doherty GM, Haugen BR, Kloos RT, Lee SL, Mandel SJ, Mazzaferri EL, McIver B, et al: Revised American Thyroid Association management guidelines for patients with thyroid nodules and differentiated thyroid cancer**.** Thyroid 2009, 19**:**1167–1214. 10.1089/thy.2009.0110 19860577

[22] Durante C, Haddy N, Baudin E, Leboulleux S, Hartl D, Travagli JP, Caillou B, Ricard M, Lumbroso JD, De Vathaire F, Schlumberger M (2006). Long-term outcome of 444 patients with distant metastases from papillary and follicular thyroid carcinoma: benefits and limits of radioiodine therapy. J Clin Endocrinol Metab.

[23] Pasca di Magliano M, Di Lauro R, Zannini M (2000). Pax8 has a key role in thyroid cell differentiation. Proc Natl Acad Sci U S A.

[24] Fernandez LP, Lopez-Marquez A, Santisteban P (2015). Thyroid transcription factors in development, differentiation and disease. Nat Rev Endocrinol.

[25] Ohno M, Zannini M, Levy O, Carrasco N, di Lauro R (1999). The paired-domain transcription factor Pax8 binds to the upstream enhancer of the rat sodium/iodide symporter gene and participates in both thyroid-specific and cyclic-AMP-dependent transcription. Mol Cell Biol.

[26] Dai G, Levy O, Carrasco N (1996). Cloning and characterization of the thyroid iodide transporter. Nature.

[27] Cancer Genome Atlas Research N (2014). Integrated genomic characterization of papillary thyroid carcinoma. Cell.

[28] Waltz F, Pillette L, Ambroise Y (2010). A nonradioactive iodide uptake assay for sodium iodide symporter function. Anal Biochem.

[29] Sewell W, Reeb A, Lin RY. An orthotopic mouse model of anaplastic thyroid carcinoma. J Vis Exp. 2013. 10.3791/50097 PMC3664970 23628990

[30] Shin G, Kang TW, Yang S, Baek SJ, Jeong YS, Kim SY (2011). GENT: gene expression database of normal and tumor tissues. Cancer Inform.

[31] Weinstein JN, Collisson EA, Mills GB, Shaw KR, Ozenberger BA, Ellrott K, Shmulevich I, Sander C, Stuart JM, Cancer Genome Atlas Research N (2013). The Cancer genome atlas pan-Cancer analysis project. Nat Genet.

[32] Kleiman DA, Buitrago D, Crowley MJ, Beninato T, Veach AJ, Zanzonico PB, Jin M, Fahey TJ, Zarnegar R (2013). Thyroid stimulating hormone increases iodine uptake by thyroid cancer cells during BRAF silencing. J Surg Res.

[33] Liu DX, Hu SY, Hou P, Jiang D, Condouris S, Xing MZ (2007). Suppression of BRAF/MEK/MAP kinase pathway restores expression of iodide-metabolizing genes in thyroid cells expressing the V600E BRAF mutant. Clin Cancer Res.

[34] Schlumberger MJ (1998). Papillary and follicular thyroid carcinoma. N Engl J Med.

[35] Rosignolo F, Sponziello M, Durante C, Puppin C, Mio C, Baldan F, Di Loreto C, Russo D, Filetti S, Damante G (2016). Expression of PAX8 target genes in papillary thyroid carcinoma. PLoS One.

[36] Lewis BP, Burge CB, Bartel DP (2005). Conserved seed pairing, often flanked by adenosines, indicates that thousands of human genes are microRNA targets. Cell.

[37] Wong N, Wang XW (2015). miRDB: an online resource for microRNA target prediction and functional annotations. Nucleic Acids Res.

[38] John B, Enright AJ, Aravin A, Tuschl T, Sander C, Marks DS (2004). Human MicroRNA targets. PLoS Biol.

[39] Guo HL, Ingolia NT, Weissman JS, Bartel DP (2010). Mammalian microRNAs predominantly act to decrease target mRNA levels. Nature.

[40] Lesinski GB (2013). The potential for targeting the STAT3 pathway as a novel therapy for melanoma. Future Oncol.

[41] Croker BA, Kiu H, Nicholson SE (2008). SOCS regulation of the JAK/STAT signalling pathway. Semin Cell Dev Biol.

[42] Ricarte-Filho JC, Ryder M, Chitale DA, Rivera M, Heguy A, Ladanyi M, Janakiraman M, Solit D, Knauf JA, Tuttle RM (2009). Mutational profile of advanced primary and metastatic radioactive iodine-refractory thyroid cancers reveals distinct Pathogenetic roles for BRAF, PIK3CA, and AKT1. Cancer Res.

[43] Riesco-Eizaguirre G, Gutierrez-Martinez P, Garcia-Cabezas MA, Nistal M, Santisteban P (2006). The oncogene BRAFV600E is associated with a high risk of recurrence and less differentiated papillary thyroid carcinoma due to the impairment of Na+/I- targeting to the membrane. Endocr Relat Cancer.

[44] Giordano TJ, Kuick R, Thomas DG, Misek DE, Vinco M, Sanders D, Zhu ZW, Ciampi R, Roh M, Shedden K (2005). Molecular classification of papillary thyroid carcinoma: distinct BRAF, RAS, and RET/PTC mutation-specific gene expression profiles discovered by DNA microarray analysis. Oncogene.

[45] Di Palma T, Filippone MG, Pierantoni GM, Fusco A, Soddu S, Zannini M. Pax8 has a critical role in epithelial cell survival and proliferation. Cell Death Dis. 2013;4. 10.1038/cddis.2013.262 PMC3730432 23868062

[46] Postiglione MP, Parlato R, Rodriguez-Mallon A, Rosica A, Mithbaokar P, Maresca M, Marians RC, Davies TF, Zannini MS, De Felice M, Di Lauro R (2002). Role of the thyroid-stimulating hormone receptor signaling in development and differentiation of the thyroid gland. Proc Natl Acad Sci U S A.

