# Supplementary material for: Low dose radiation regulates BRAF-induced thyroid cellular dysfunction and transformation
Source: Cell Commun Signal. 2019 Feb 13;17:12. doi: 10.1186/s12964-019-0322-x (PMC6373124; doi:10.1186/s12964-019-0322-x)

**Low dose radiation regulates BRAF-induced thyroid cellular dysfunction and transformation**

Neha Kaushik^1,8^, Min-Jung Kim^2,8^, Nagendra Kumar Kaushik^3^, Jae Kyung Myung^4^, Mi-Young Choi^1^, Jae-Hyeok Kang^1^, Hyuk-Jin Cha^5^, Cha-Soon Kim^6^, Seon-Young Nam^7*^, Su-Jae Lee^1*^

*^1^Department of Life Science, Research Institute for Natural Sciences, Hanyang University, Seoul 04763, Republic of Korea*

*^2^Laboratory of Radiation Exposure and Therapeutics, National Radiation Emergency Medical Center, Korea Institute of Radiological and Medical Sciences, Seoul, Korea.*

*^3^Plasma Bioscience Research Center, Applied Plasma Medicine Center, Department of Electrical and Biological Physics, Kwangwoon University, Seoul, 01897, Republic of Korea*

*^4^Department of Radiation Pathology, Korea Cancer Center Hospital, Seoul, Korea*

*^5^* *College of Pharmacy, Seoul National University, Seoul, Korea*

*^6^Department of Preventive medicine, College of Medicine, Dongguk University, Gyeongju 38066, Korea*

*^7^Radiation Health Institute, Korea Hydro and Nuclear Power Co. Ltd, Seoul, Korea*

*^8^These authors equally contributed to this work.*

**Running title:** LDR recovers BRAF suppressive target genes.

***Correspondence should be addressed to:** Su-Jae Lee, PhD**.** Professor**,** Laboratory of Molecular Biochemistry, Department of Life Science**,** Hanyang University**,** 17 Haengdang-Dong, Seongdong-Ku, Seoul 04763, Korea**.** Phone: 82-2-2220-2557, Fax: 82-2-2299-0762**.** E-mail: [sj0420@hanyang.ac.kr](mailto:sj0420@hanyang.ac.kr) (S.J.L.) or Co-corresponding: Dr. Seon-Young Nam, Radiation Health Institute, Korea Hydro and Nuclear Power Co. Ltd, Seoul, Korea. Email: [namsy6660@khnp.co.kr](mailto:namsy6660@khnp.co.kr) (S.Y.N.)

All authors Email addresses:

Neha Kaushik: [neha.bioplasma@gmail.com](mailto:neha.bioplasma@gmail.com)

Min-Jung Kim: [kimmj74@kirams.re.kr](mailto:kimmj74@kirams.re.kr)

Nagendra Kumar Kaushik: [kaushik.nagendra@gmail.com](mailto:kaushik.nagendra@gmail.com)

Jae Kyung Myung: [tontos016@kirams.re.kr](mailto:tontos016@kirams.re.kr)

Mi-Young Choi: [keith0323@naver.com](mailto:keith0323@naver.com)

Jae-Hyeok Kang: [jaehyeok1121@gmail.com](mailto:jaehyeok1121@gmail.com)

Hyuk-Jin Cha: [hjcha93@snu.ac.kr](mailto:hjcha93@snu.ac.kr)

Cha-Soon Kim: bovine1@daum.net

Seon-Young Nam: [namsy6660@khnp.co.kr](mailto:namsy6660@khnp.co.kr)

Su-Jae Lee: [sj0420@hanyang.ac.kr](mailto:sj0420@hanyang.ac.kr)

**Additional file 1: Figure S1. Low dose radiation reduces cellular growth in BRAF-transformed thyroid cells.** (A) Cellular growth was accessed in normal BRAF^V600E^ transformed N-thy ori-3-1 and BRAF^V600E^-mutated 850-5C and BCPAP thyroid cancer cells after low dose radiation (LDR) exposure. (B) Thyroid stimulating hormone (TSHR) ELISA assay in normal BRAF^V600E^ transformed N-thy ori-3-1 after low dose radiation (LDR) exposure. ß-actin was used as a control for normalization. **p* < 0.05, ***p* < 0.001, and ****p* < 0.0001.


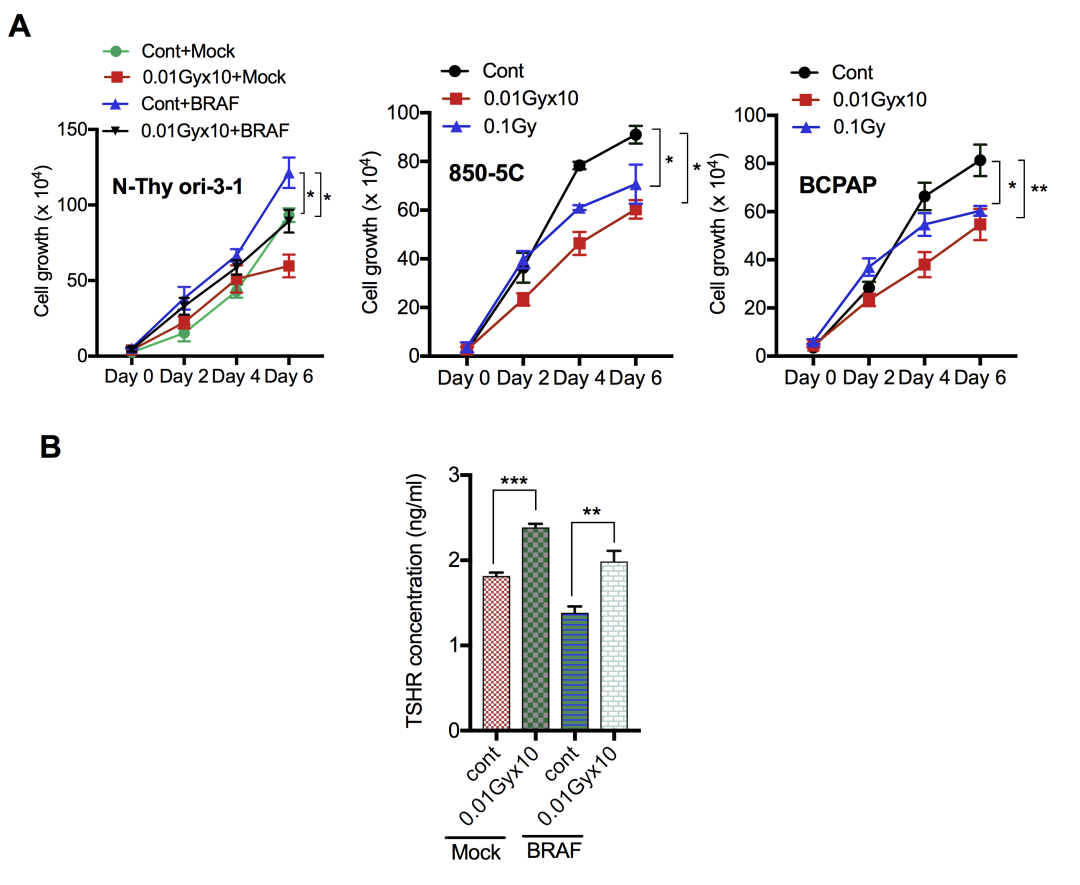


**Additional file 1: Figure S2. High dose radiation increases thyroid metabolizing gene levels in BRAF-transformed thyroid cells.** (A) qPCR analysis for *PAX8*, *NIS* and *TG* mRNA expression in normal BRAF^V600E^ transformed N-thy ori-3-1 thyroid cells after 2Gy of High dose radiation (HDR). (B) qPCR analysis for *PAX8*, *NIS*, *TG*, *TTF-1* and *TTF-2* mRNA expression in BRAF^V600E^ mutated BCPAP thyroid carcinoma cells. *ß-actin* was used as a control for normalization. **p* < 0.05, ***p* < 0.001, and ****p* < 0.0001.

**Additional file 1: Figure S3. HDR increases PAX8-targeting miRNAs in BRAF-mutated thyroid cancer cells.** (A) Confirmation of anti-miR-144-3p and anti-miR-330-5p inhibitors expression in 850-5C cancer cells using qPCR analysis. (B) qPCR analysis for miR-144 and miR-330-5p levels in BRAF^V600E^ mutated 850-5C and BCPAP thyroid cancer cells after HDR treatment at a dose of 2Gy. (C) Expression of miR-330-5p mimics was confirmed in BCPAP cells. (D) Suppressors of cytokine signaling (SOCS) levels were analyzed in BCPAP thyroid cancer cells after LDR treatment at fractionated as well as single dose. ß-actin was used as a control for normalization. **p* < 0.05, ***p* < 0.001, and ****p* < 0.0001.


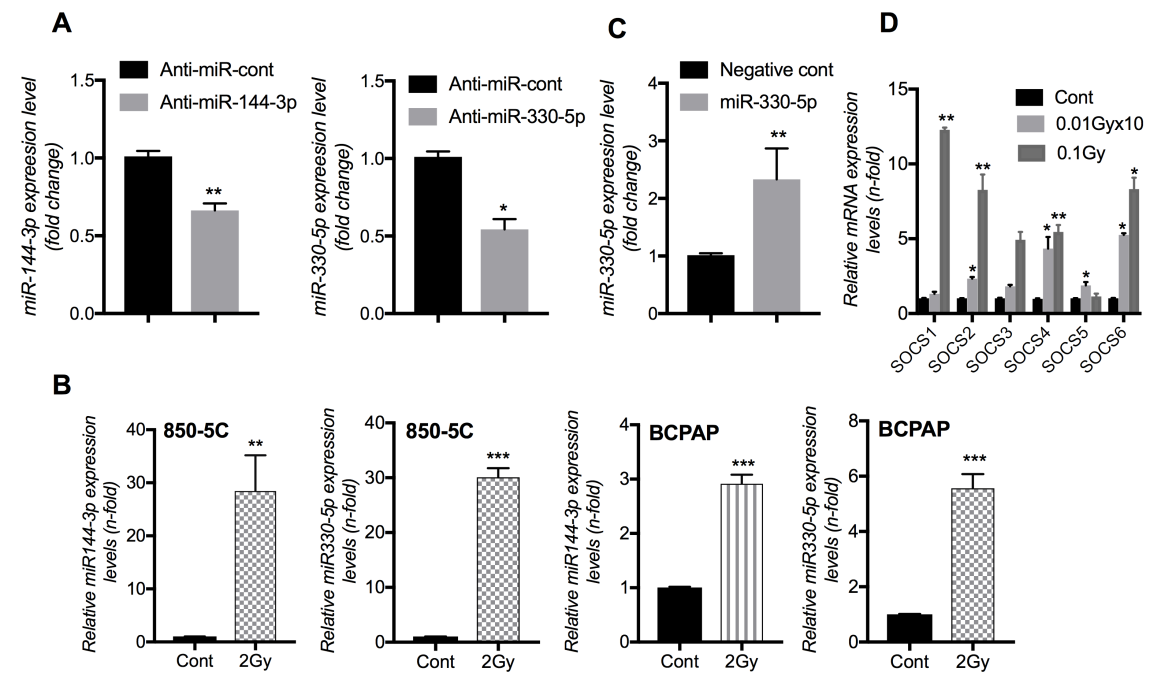

Supplement: Supplementary file 1 — Figures S1-S3. (DOCX 612 kb) [file 12964_2019_322_MOESM1_ESM.docx]
